# Response to PD1 inhibition in conventional chondrosarcoma

**DOI:** 10.1186/s40425-018-0413-z

**Published:** 2018-09-25

**Authors:** Michael J. Wagner, Robert W. Ricciotti, Jose Mantilla, Elizabeth T. Loggers, Seth M. Pollack, Lee D. Cranmer

**Affiliations:** 1 Division of Medical Oncology, University of Washington School of Medicine, 825 Eastlake Avenue E, Seattle, WA 98109 USA; 2 Clinical Research Division, Fred Hutchinson Cancer Research Center, 1100 Fairview Ave N, Seattle, WA 98109 USA; 3 Department of Pathology, University of Washington School of Medicine, 1959 NE Pacific St, Seattle, WA 98195 USA

**Keywords:** Chondrosarcoma, Immunotherapy, Sarcoma, Bone cancer

## Abstract

**Background:**

Chondrosarcoma is one of the most common malignant bone tumors in adults. Conventional chondrosarcoma represents around 85% of all chondrosarcomas and is notoriously difficult to treat with chemotherapy.

**Case presentation:**

We describe a 67-year-old man with metastatic conventional chondrosarcoma who was treated with nivolumab. Treatment was discontinued after restaging showed increased tumor burden, which later proved to be pseudoprogression. The patient restarted nivolumab and continues to have a near complete response.

**Conclusion:**

Conventional chondrosarcoma may be sensitive to checkpoint inhibitors. Further, this case demonstrates clearly the phenomenon of pseudo-progression in this disease, a factor that must be considered in the design of clinical trials and clinical care. This case supports additional study of immunomodulatory agents in this deadly disease.

## Background

Chondrosarcoma is one of the most common malignant bone tumors in adults [1]. Chondrosarcomas are further stratified into conventional, mesenchymal, dedifferentiated, and clear cell subtypes. Conventional chondrosarcoma represents around 85% of all chondrosarcomas and is notoriously difficult to treat with chemotherapy [1]. Mesenchymal chondrosarcoma is generally treated with Ewing sarcoma chemotherapy regimens, and dedifferentiated chondrosarcoma is treated as osteosarcoma. [1] Current guidelines of the National Comprehensive Cancer Network (NCCN, version 1.2018) indicate that “conventional chondrosarcoma (Grades 1-3) has no known standard chemotherapy options”. Any efficacious systemic treatment would expand our current armamentarium for this difficult disease.

Immunotherapy has had enormous success in treating multiple cancer subtypes. Particular success has been seen with immune checkpoint inhibitors, which are now approved as standard therapy in melanoma [2, 3], lung [4], genitourinary [5, 6], and cancers with microsatellite instability [7] with an increasing number of indications as new data emerges. Initial studies in sarcoma have had mixed results. [8, 9] An early study of ipilimumab in synovial sarcoma was stopped due to lack of responses and poor accrual [10]. To date, little is understood about which sarcoma subtypes are most susceptible to immunotherapy and what drives the responses seen. Some of the studies of checkpoint inhibitors in sarcoma have included mesenchymal [11] and dedifferentiated chondrosarcomas [8, 11] with variable results, but these tumors are distinct from conventional chondrosarcoma in their general responsiveness to chemotherapy. [1] Conventional chondrosarcoma patients were not included in these studies.

Here we report a case of conventional chondrosarcoma with a near complete response after pseudo-progression on a checkpoint inhibitor. To our knowledge, this represents the first reported case of a dramatic response of a conventional chondrosarcoma to immunotherapy.

## Case presentation

**Fig. 1:**
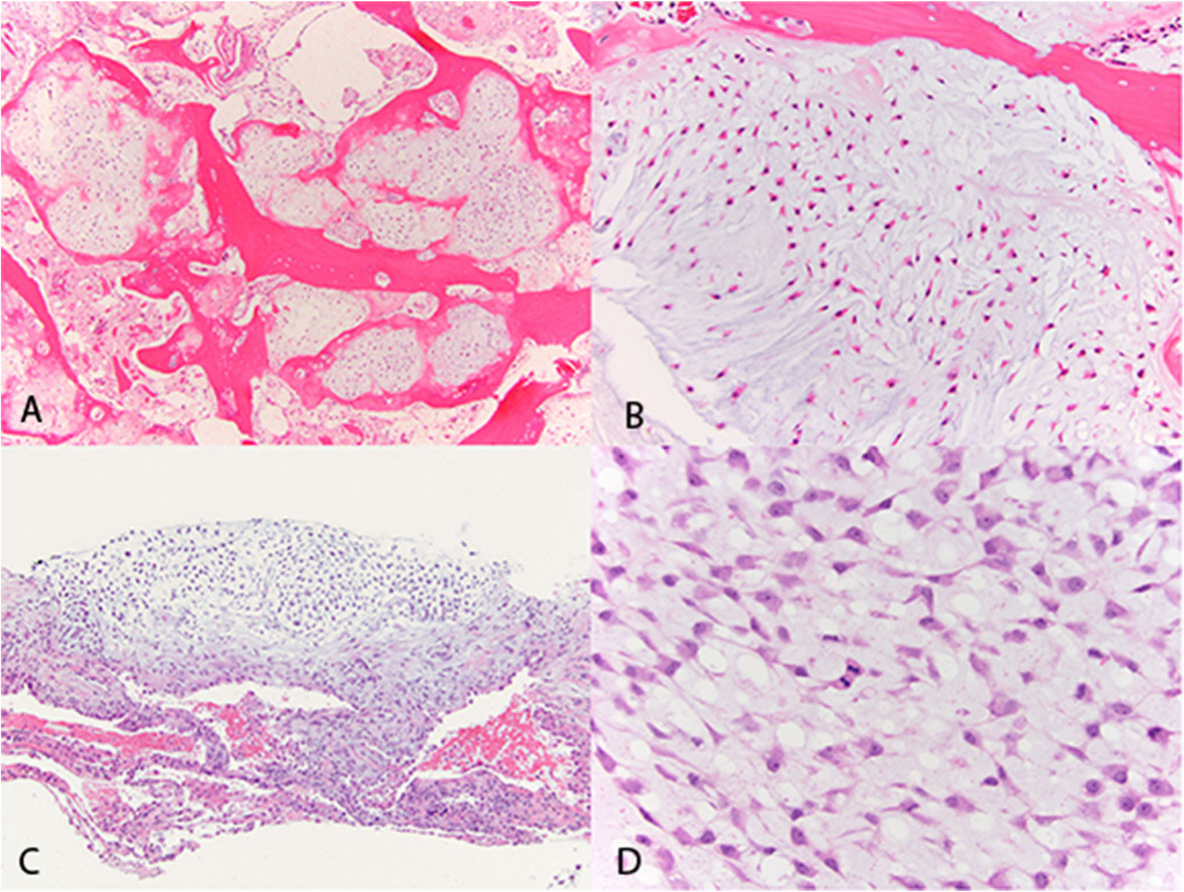
Hematoxylin and eosin stained slides of the primary tumor at 40X magnification (**a**) and 100X magnification (**b**) and lung metastasis at 40X magnification (**c**) and 200X magnification (**d**)

The patient is a 67-year-old man with a history of localized prostate cancer treated with prostatectomy. He initially presented with a 22 cm lytic mass of the distal femur. Core needle biopsy revealed grade 2 conventional chondrosarcoma. After resection, final pathology showed grade 3 conventional chondrosarcoma (Fig. 1a-b).

**Fig. 2:**
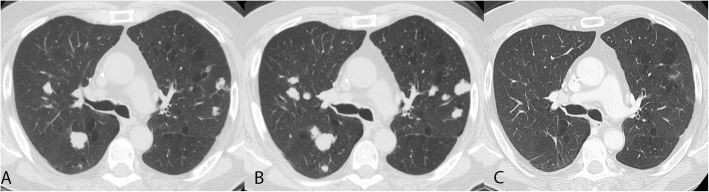
Computerized tomography (CT) images of lung nodules. CT at time of initiation of nivolumab (**a**), 2 months after initiation of nivolumab (**b**) and 6 months after initiation of nivolumab (**c**)

Nine months later, he developed innumerable, biopsy-proven pulmonary metastases (Fig. 1c-d and Fig. 2a). Due to the general lack of efficacy of cytotoxic chemotherapy for conventional chondrosarcoma [1], the patient’s inability to travel to participate in clinical trials and following extensive discussion with the patient, he initiated nivolumab 240 mg (flat dose) intravenously every 2 weeks on a compassionate use basis. After 4 doses, the pulmonary nodules increased in size and number (Fig. 2b). Therapy was stopped, with plans to enroll in a clinical trial that was not available at our site at the time of nivolumab initiation.

Three months later, pre-trial CT scans revealed a near complete response in his pulmonary nodules, with most nodules resolving. One remaining index nodule previously measuring 16x17mm decreased to 5 mm and another measuring 11 mm decreased to 4 mm. Rather than enrolling on trial, he reinitiated nivolumab therapy and continues with improvement in his few remaining pulmonary nodules (Fig. 2c).

We conducted analyses to understand the underlying pathogenetic mechanisms operative in this case. NextGen sequencing (NGS) revealed a mutation in exon 4 of *IDH2* and a variant of unknown significance in exon 11 of *BRCA2.* The tumor was microsatellite stable by sequencing and demonstrated no loss of expression of mismatch repair proteins (MLH1, MSH2, MSH6, and PMS2) when assessed by immunohistochemistry. PTEN expression was retained. The tumor did not express PD-1 and was 1% positive (2+) for PD-L1. Tumor mutational burden was low (4 mutations/Mb).

## Discussion and conclusions

Conventional chondrosarcomas are resistant to radiotherapy and chemotherapy. Because of this, there is no defined standard of care treatment for patients with unresectable or metastatic disease. Several mechanisms of have been proposed to explain the chemoresistance of the disease. Chondrosarcoma cell lines expressing MDR1 and P-glycoprotein are associated with anthracycline resistance. [12] Additional experiments link BCL-2 expression and BCL-2-mediated resistance to apoptosis in the presence of chemotherapy. [13] The relative resistance of conventional chondrosarcoma is also attributed to the poor vascularity and high deposition of extracellular matrix in the tumors, and their relatively slow rate of growth. [14]

Efforts to identify druggable targets in chondrosarcoma have recently revealed recurrent mutations in *IDH1* and *IDH2*, as was seen in the patient presented here. These are almost always found in chondrosarcoma cases associated with the Maffucci and Ollier syndromes [15], and in about half of other chondrosarcomas. [16] IDH is an enzyme in the Krebs cycle that normally catalyzes the conversion of isocitrate into alpha-ketoglutarate. Mutated IDH is able to catalyze conversion of α-ketoglutarate into delta-2-hydroxyglutarate (2HG). Mutant *IDH2* leads to increased intracellular 2HG and hypermethylated DNA in mesenchymal cells, inhibiting their differentiation in a manner reversible by treatment with DNA-hypomethylating agents. [17] There are conflicting data regarding the antitumor effects of direct *IDH1* inhibition in chondrosarcoma. [18, 19] The clinical utility of this approach is being tested in trials for patients with IDH mutated cancers including chondrosarcoma (NCT02073994, NCT02746081). Interestingly, introduction of an activating *IDH2* mutation in a syngeneic mouse model of glioma led to reduced levels of CXCL10 and suppression of cytotoxic T-cell recruitment to the tumor. [20] *IDH* mutant gliomas also escape natural killer cell mediated lysis by epigenetic reprogramming that leads to downregulation of NKG2D ligand expression [21]. Given these findings in glioma, one might have expected this *IDH2* mutant chondrosarcoma to evade the immune system. However, this patient responded in spite of the presence of the *IDH2* mutation.

Other oncogenic pathways being studied as potential targets for chondrosarcoma include the PI3K-Akt-mTOR pathway, SRC pathway, and hedgehog pathway . [22] A small retrospective series of chondrosarcoma patients suggested clinical benefit with VEGFR2 inhibitors. [23] Additional efforts to identify targets by NGS have revealed recurrent alterations in TP53, ACVR2A, COL2A1, and YEATS2 in addition to the previously identified recurrent *IDH* mutations. [24]

Immunotherapy agents are increasingly demonstrating success in many cancer subtypes, and there have been preclinical suggestions that they may work in chondrosarcoma. An early report demonstrated that tumor specific immune responses against chondrosarcoma antigens is possible. [25] Cancer testis antigens (CTAs) such as NY-ESO-1, LAGE-1 s and PRAME are expressed in some sarcomas and may represent cancer-specific antigens to be used as targets for immunotherapies. A subset of chondrosarcomas express NY-ESO-1 or LAGE-1 s at baseline, and CTA expression is upregulated in chondrosarcoma cell lines after treatment with decitabine. [26] HMW-MAA is expressed in about 48% of chondrosarcomas and represents another potential antigen target for T-cells. [27] MAGE-A family CTAs are also expressed in chondrosarcoma [28] and can elicit lysis by cytotoxic T-lymphocytes. [29] In a rat model, depletion of intratumoral cytotoxic T-lymphocytes led to increased rates of tumor growth. [30] Collectively, these data suggest a role for immunomodulatory agents in chondrosarcoma.

In published clinical studies of immune checkpoint inhibitors in sarcoma, objective responses were seen in 2 patients with dedifferentiated chondrosarcoma. [8, 11] To our knowledge no published reports have included conventional chondrosarcoma patients treated with checkpoint inhibitors. One might consider a prospective study of single agent PD1 inhibition using immune criteria for response assessment. [31] The clear clinical benefit in this patient demonstrates that conventional chondrosarcoma may be sensitive to checkpoint inhibitors, and supports additional study of immunomodulatory agents in this disease. Further, this case demonstrates clearly the phenomenon of pseudo-progression in this disease, a factor that must be considered in the design of any clinical trials and clinical care.

## Abbreviations

CTA: Cancer testis antigen
NGS: NextGen sequencing
